# Supplementary material for: Molecular Pathogenesis and Regulation of the miR-29-3p-Family: Involvement of ITGA6 and ITGB1 in Intra-Hepatic Cholangiocarcinoma
Source: Cancers (Basel). 2021 Jun 4;13(11):2804. doi: 10.3390/cancers13112804 (PMC8200054; doi:10.3390/cancers13112804)
Supplement: Supplementary file 1 [file cancers-13-02804-s001.zip › supplementary files/Figure S2A.pptx]

## Slide 1
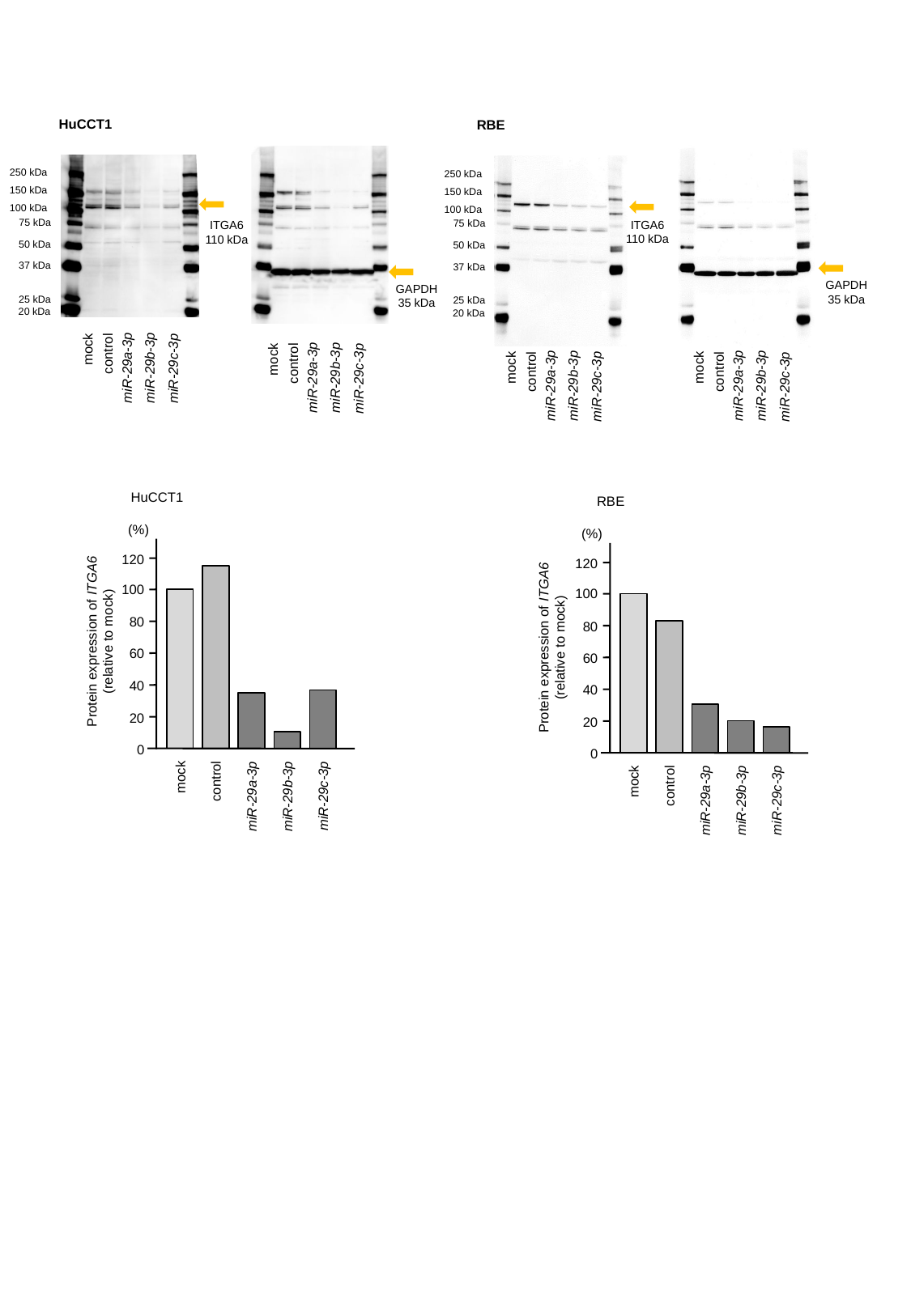

HuCCT1
RBE
250 kDa
150 kDa
100 kDa
　75 kDa
　50 kDa
　37 kDa
　25 kDa
　20 kDa
250 kDa
150 kDa
100 kDa
　75 kDa
　50 kDa
　37 kDa
　25 kDa
　20 kDa
ITGA6
110 kDa
ITGA6
110 kDa
GAPDH
35 kDa
GAPDH
35 kDa
mock
control
miR-29a-3p
miR-29b-3p
miR-29c-3p
mock
control
miR-29a-3p
miR-29b-3p
miR-29c-3p
mock
control
miR-29a-3p
miR-29b-3p
miR-29c-3p
mock
control
miR-29a-3p
miR-29b-3p
miR-29c-3p
HuCCT1
(%)
120
100
80
60
40
20
0
control
mock
Protein expression of ITGA6
(relative to mock)
miR-29c-3p
miR-29a-3p
miR-29b-3p
RBE
(%)
120
100
80
60
40
20
0
mock
control
Protein expression of ITGA6
(relative to mock)
miR-29a-3p
miR-29b-3p
miR-29c-3p
