# Supplementary material for: Molecular Pathogenesis and Regulation of the miR-29-3p-Family: Involvement of ITGA6 and ITGB1 in Intra-Hepatic Cholangiocarcinoma
Source: Cancers (Basel). 2021 Jun 4;13(11):2804. doi: 10.3390/cancers13112804 (PMC8200054; doi:10.3390/cancers13112804)
Supplement: Supplementary file 1 [file cancers-13-02804-s001.zip › supplementary files/Figure S4.pptx]

## Slide 1
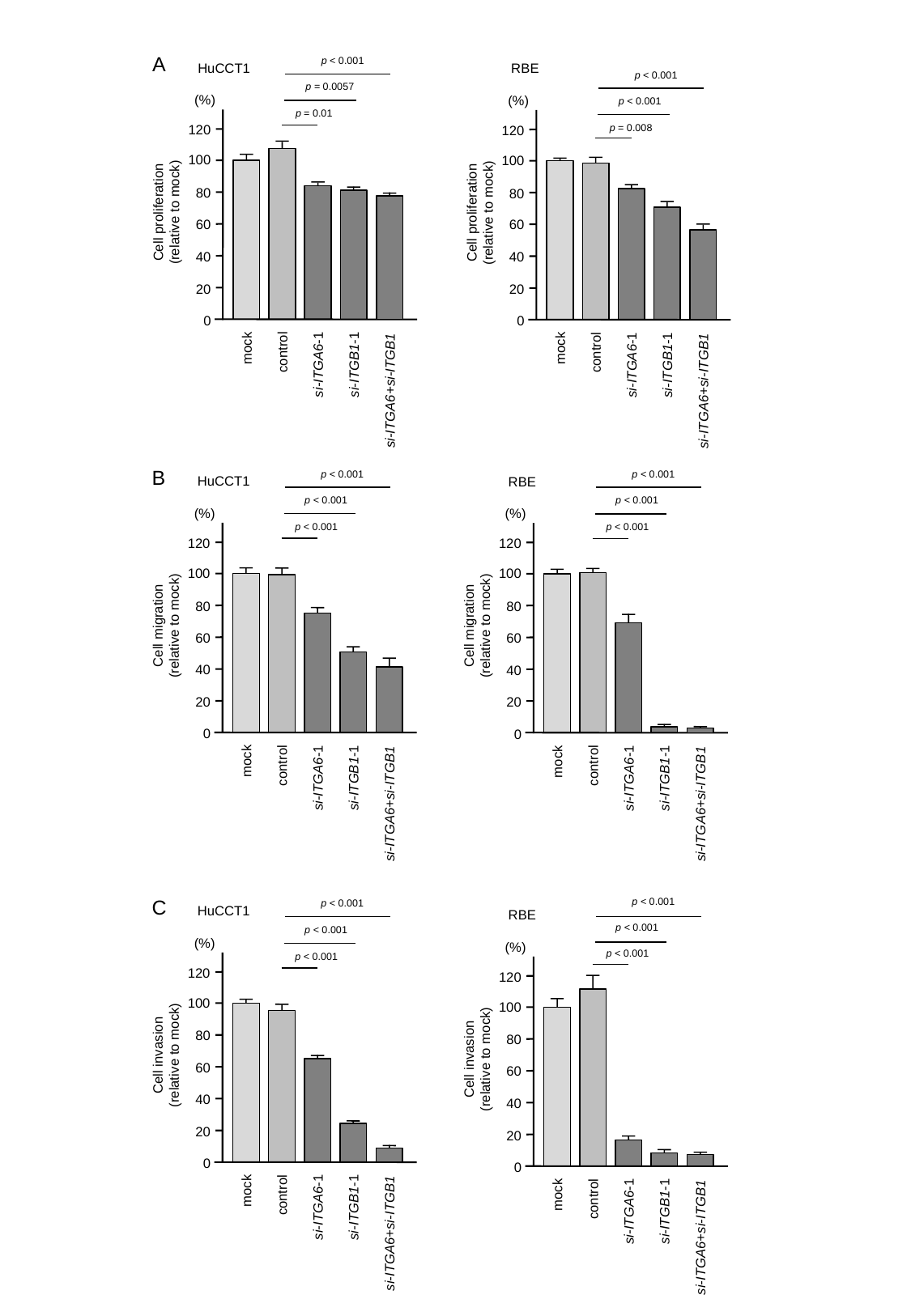

A
HuCCT1
p = 0.0057
p = 0.01
(%)
120
control
100
mock
80
si-ITGA6-1
si-ITGB1-1
Cell proliferation
(relative to mock)
si-ITGA6+si-ITGB1
60
40
20
0
p < 0.001
RBE
(%)
p < 0.001
p = 0.008
120
100
control
mock
80
si-ITGA6-1
Cell proliferation
(relative to mock)
si-ITGB1-1
60
si-ITGA6+si-ITGB1
40
20
0
p < 0.001
B
HuCCT1
p < 0.001
p < 0.001
(%)
120
100
mock
control
80
Cell migration
(relative to mock)
si-ITGA6-1
60
si-ITGB1-1
40
si-ITGA6+si-ITGB1
20
0
p < 0.001
p < 0.001
RBE
p < 0.001
p < 0.001
(%)
120
100
control
mock
80
Cell migration
(relative to mock)
si-ITGA6-1
60
40
20
0
si-ITGB1-1
si-ITGA6+si-ITGB1
C
HuCCT1
p < 0.001
p < 0.001
(%)
120
100
mock
control
80
Cell invasion
(relative to mock)
60
si-ITGA6-1
40
20
si-ITGB1-1
si-ITGA6+si-ITGB1
0
p < 0.001
p < 0.001
RBE
p < 0.001
p < 0.001
(%)
120
control
100
mock
80
Cell invasion
(relative to mock)
60
40
20
si-ITGA6-1
si-ITGB1-1
si-ITGA6+si-ITGB1
0
