# Supplementary material for: Molecular Pathogenesis and Regulation of the miR-29-3p-Family: Involvement of ITGA6 and ITGB1 in Intra-Hepatic Cholangiocarcinoma
Source: Cancers (Basel). 2021 Jun 4;13(11):2804. doi: 10.3390/cancers13112804 (PMC8200054; doi:10.3390/cancers13112804)
Supplement: Supplementary file 1 [file cancers-13-02804-s001.zip › supplementary files/Figure S5.pptx]

## Slide 1
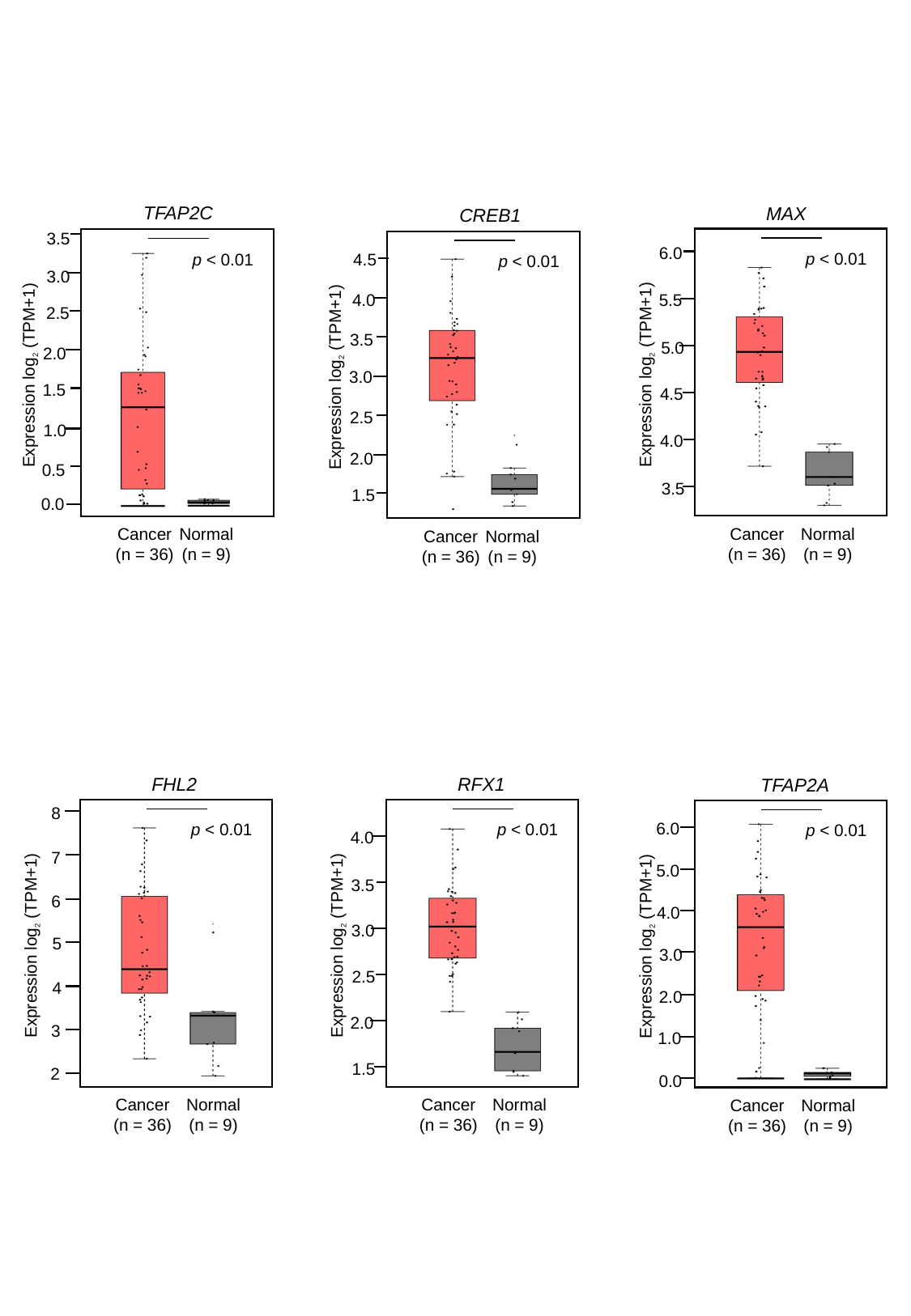

TFAP2C
3.5
p < 0.01
3.0
2.5
2.0
Expression log2 (TPM+1)
1.5
1.0
0.0
Cancer
(n = 36)
Normal
(n = 9)
0.5
MAX
6.0
p < 0.01
5.5
5.0
Expression log2 (TPM+1)
4.5
4.0
3.5
Cancer
(n = 36)
Normal
(n = 9)
CREB1
4.5
p < 0.01
4.0
3.5
3.0
Expression log2 (TPM+1)
2.5
2.0
1.5
Cancer
(n = 36)
Normal
(n = 9)
FHL2
8
p < 0.01
7
6
5
Expression log2 (TPM+1)
4
3
2
Cancer
(n = 36)
Normal
(n = 9)
RFX1
p < 0.01
4.0
3.5
3.0
Expression log2 (TPM+1)
2.5
2.0
1.5
Cancer
(n = 36)
Normal
(n = 9)
TFAP2A
6.0
p < 0.01
5.0
4.0
Expression log2 (TPM+1)
3.0
2.0
1.0
0.0
Cancer
(n = 36)
Normal
(n = 9)

## Slide 2
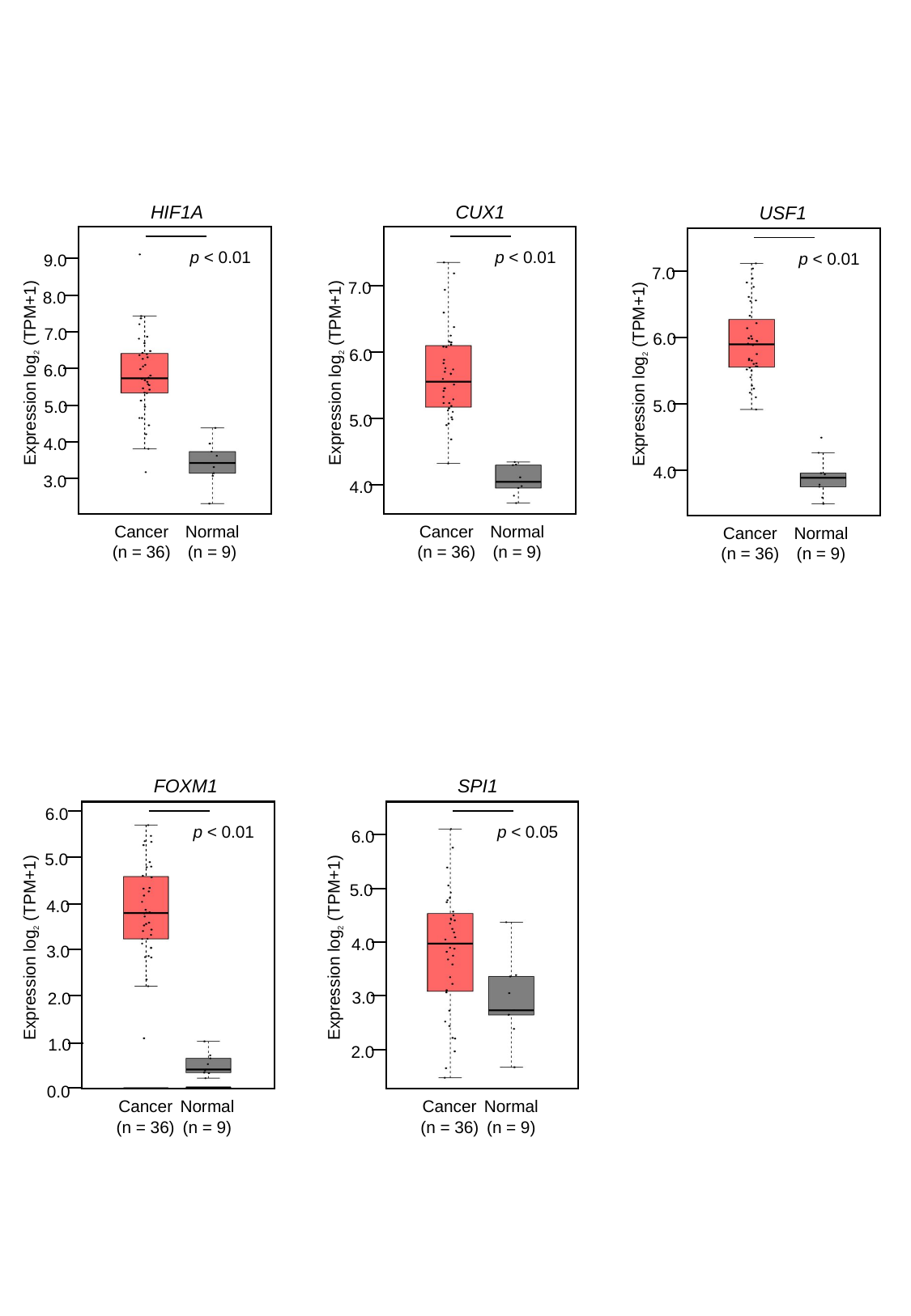

HIF1A
p < 0.01
9.0
8.0
7.0
6.0
Expression log2 (TPM+1)
5.0
4.0
3.0
Cancer
(n = 36)
Normal
(n = 9)
CUX1
p < 0.01
7.0
6.0
Expression log2 (TPM+1)
5.0
4.0
Cancer
(n = 36)
Normal
(n = 9)
USF1
p < 0.01
7.0
6.0
Expression log2 (TPM+1)
5.0
4.0
Cancer
(n = 36)
Normal
(n = 9)
FOXM1
6.0
p < 0.01
5.0
4.0
Expression log2 (TPM+1)
3.0
2.0
1.0
0.0
Cancer
(n = 36)
Normal
(n = 9)
SPI1
p < 0.05
6.0
5.0
4.0
Expression log2 (TPM+1)
3.0
2.0
Cancer
(n = 36)
Normal
(n = 9)
