# Supplementary material for: Molecular Pathogenesis and Regulation of the miR-29-3p-Family: Involvement of ITGA6 and ITGB1 in Intra-Hepatic Cholangiocarcinoma
Source: Cancers (Basel). 2021 Jun 4;13(11):2804. doi: 10.3390/cancers13112804 (PMC8200054; doi:10.3390/cancers13112804)
Supplement: Supplementary file 1 [file cancers-13-02804-s001.zip › supplementary files/Figure S6A.pptx]

## Slide 1
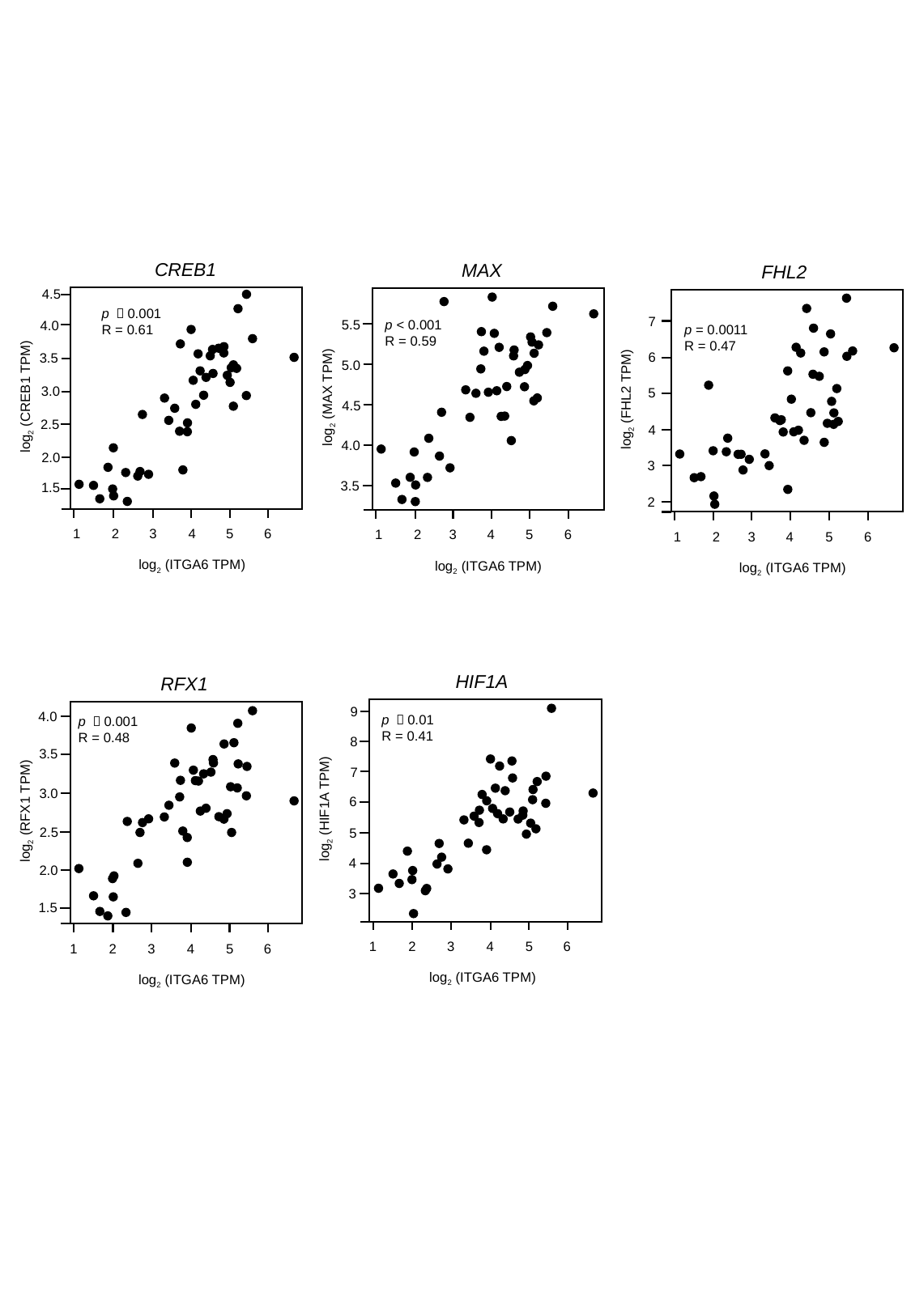

CREB1
4.5
p ＜0.001
R = 0.61
4.0
3.5
3.0
log2 (CREB1 TPM)
2.5
2.0
1.5
1
2
3
4
5
6
log2 (ITGA6 TPM)
MAX
5.5
p < 0.001
R = 0.59
5.0
log2 (MAX TPM)
4.5
4.0
3.5
1
2
3
4
5
6
log2 (ITGA6 TPM)
FHL2
7
p = 0.0011
R = 0.47
6
5
4
3
2
1
2
3
4
5
6
log2 (ITGA6 TPM)
log2 (FHL2 TPM)
HIF1A
9
p ＜0.01
R = 0.41
8
7
6
log2 (HIF1A TPM)
5
4
3
1
2
3
4
5
6
log2 (ITGA6 TPM)
RFX1
4.0
p ＜0.001
R = 0.48
3.5
3.0
log2 (RFX1 TPM)
2.5
2.0
1.5
1
2
3
4
5
6
log2 (ITGA6 TPM)
