# Supplementary material for: Molecular Pathogenesis and Regulation of the miR-29-3p-Family: Involvement of ITGA6 and ITGB1 in Intra-Hepatic Cholangiocarcinoma
Source: Cancers (Basel). 2021 Jun 4;13(11):2804. doi: 10.3390/cancers13112804 (PMC8200054; doi:10.3390/cancers13112804)
Supplement: Supplementary file 1 [file cancers-13-02804-s001.zip › supplementary files/Figure S6B.pptx]

## Slide 1
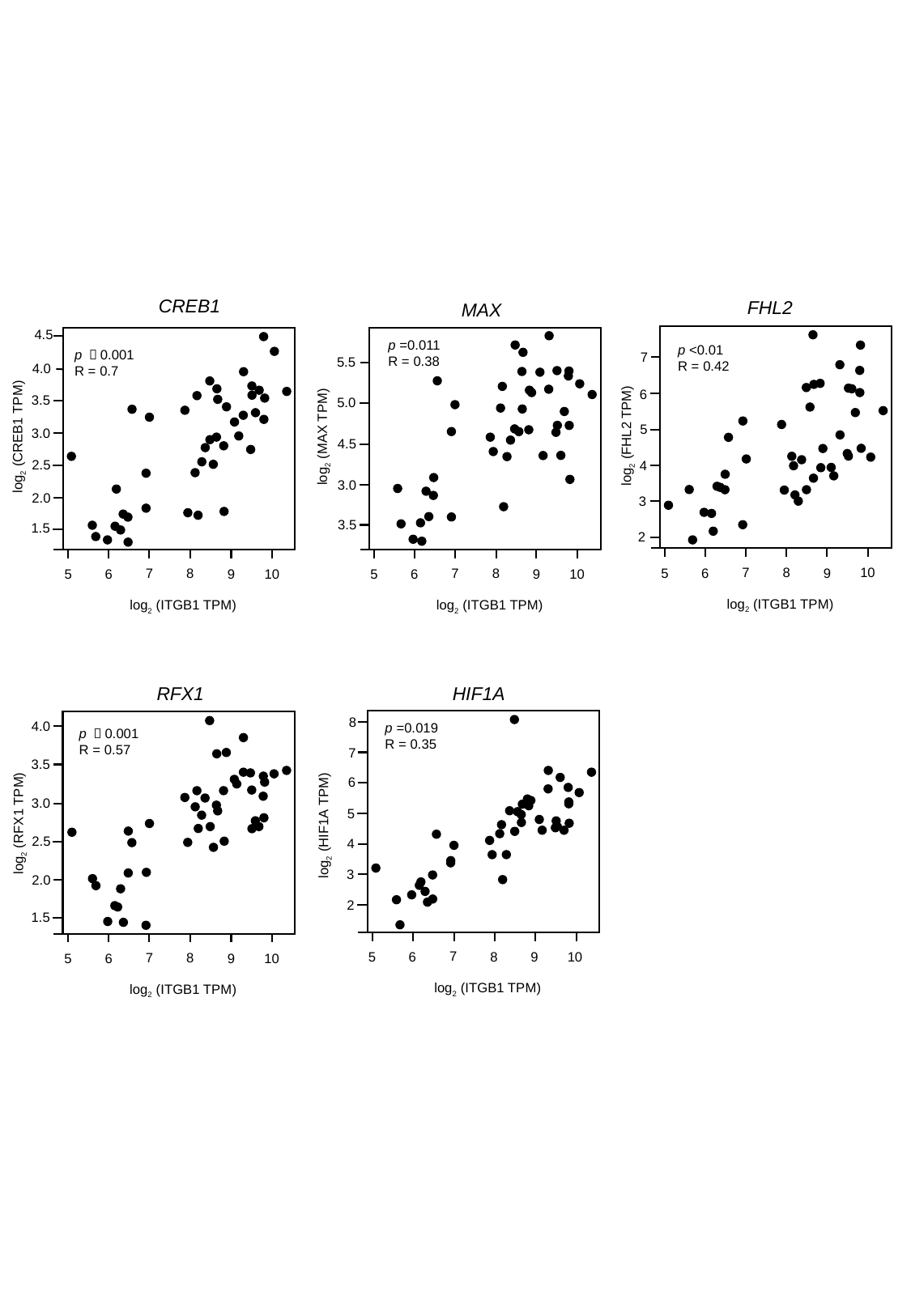

CREB1
4.5
p ＜0.001
R = 0.7
4.0
3.5
3.0
2.5
2.0
1.5
7
8
10
5
6
9
log2 (ITGB1 TPM)
FHL2
p <0.01
R = 0.42
7
6
5
4
3
2
7
8
10
5
6
9
log2 (ITGB1 TPM)
log2 (FHL2 TPM)
MAX
p =0.011
R = 0.38
5.5
5.0
4.5
3.0
3.5
7
8
10
5
6
9
log2 (ITGB1 TPM)
log2 (MAX TPM)
log2 (CREB1 TPM)
HIF1A
8
p =0.019
R = 0.35
7
6
5
log2 (HIF1A TPM)
4
3
2
7
8
10
5
6
9
log2 (ITGB1 TPM)
RFX1
4.0
p ＜0.001
R = 0.57
3.5
3.0
2.5
2.0
1.5
7
8
10
5
6
9
log2 (ITGB1 TPM)
log2 (RFX1 TPM)
