# Supplementary material for: Molecular Pathogenesis and Regulation of the miR-29-3p-Family: Involvement of ITGA6 and ITGB1 in Intra-Hepatic Cholangiocarcinoma
Source: Cancers (Basel). 2021 Jun 4;13(11):2804. doi: 10.3390/cancers13112804 (PMC8200054; doi:10.3390/cancers13112804)
Supplement: Supplementary file 1 [file cancers-13-02804-s001.zip › supplementary files/Figure S9.pptx]

## Slide 1
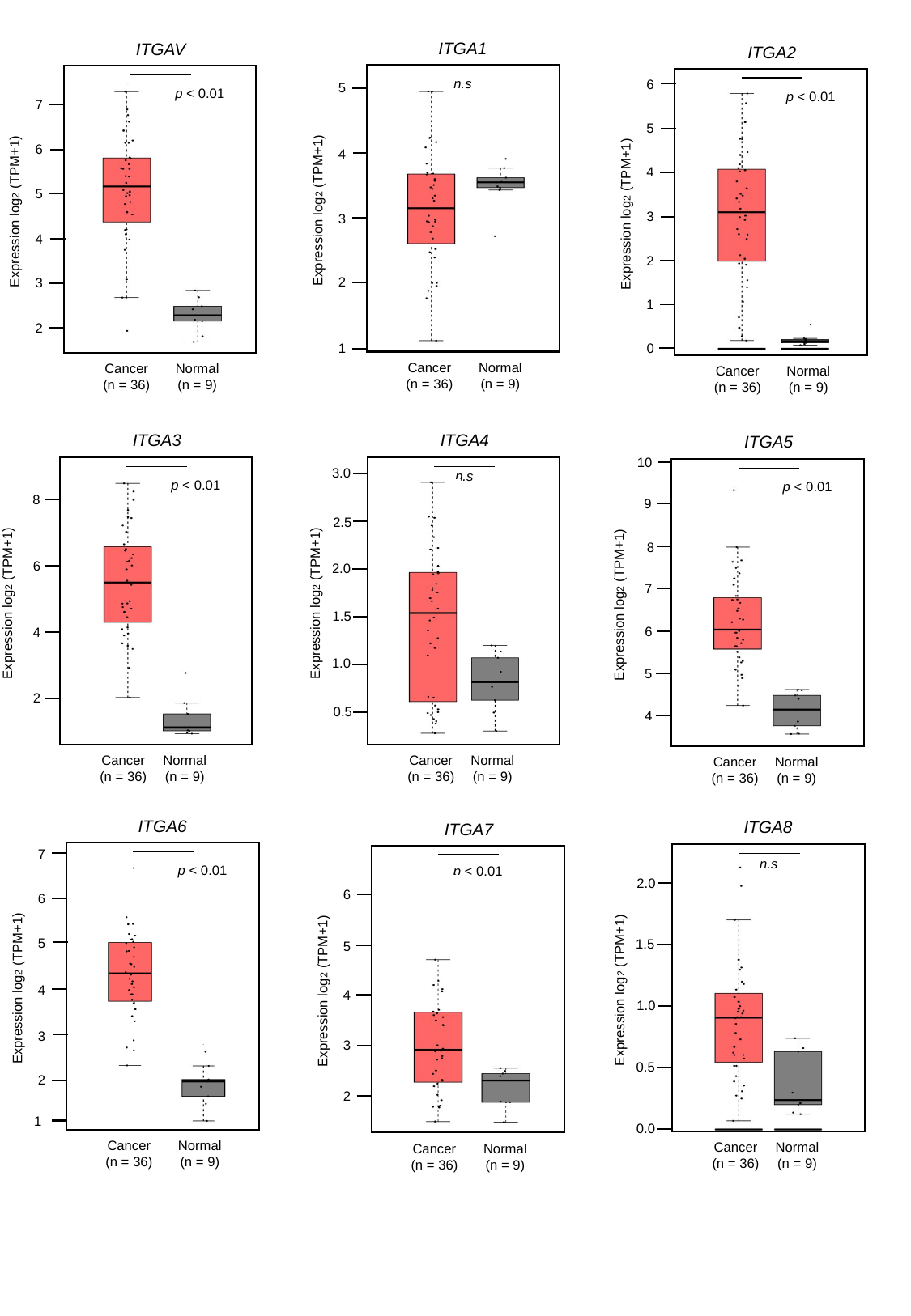

ITGA1
n.s
5
4
Expression log2 (TPM+1)
3
2
1
Cancer
(n = 36)
Normal
(n = 9)
ITGAV
p < 0.01
7
6
5
Expression log2 (TPM+1)
4
3
2
Cancer
(n = 36)
Normal
(n = 9)
ITGA2
6
p < 0.01
5
4
Expression log2 (TPM+1)
3
2
1
Cancer
(n = 36)
Normal
(n = 9)
0
ITGA3
p < 0.01
8
6
Expression log2 (TPM+1)
4
2
Cancer
(n = 36)
Normal
(n = 9)
ITGA4
3.0
2.5
2.0
Expression log2 (TPM+1)
1.5
1.0
0.5
Cancer
(n = 36)
Normal
(n = 9)
n.s
ITGA5
10
p < 0.01
9
8
7
Expression log2 (TPM+1)
6
5
4
Cancer
(n = 36)
Normal
(n = 9)
ITGA6
7
p < 0.01
6
5
Expression log2 (TPM+1)
4
3
2
1
Cancer
(n = 36)
Normal
(n = 9)
ITGA8
2.0
1.5
Expression log2 (TPM+1)
1.0
0.5
0.0
Cancer
(n = 36)
Normal
(n = 9)
n.s
ITGA7
p < 0.01
6
5
Expression log2 (TPM+1)
4
3
2
Cancer
(n = 36)
Normal
(n = 9)

## Slide 2
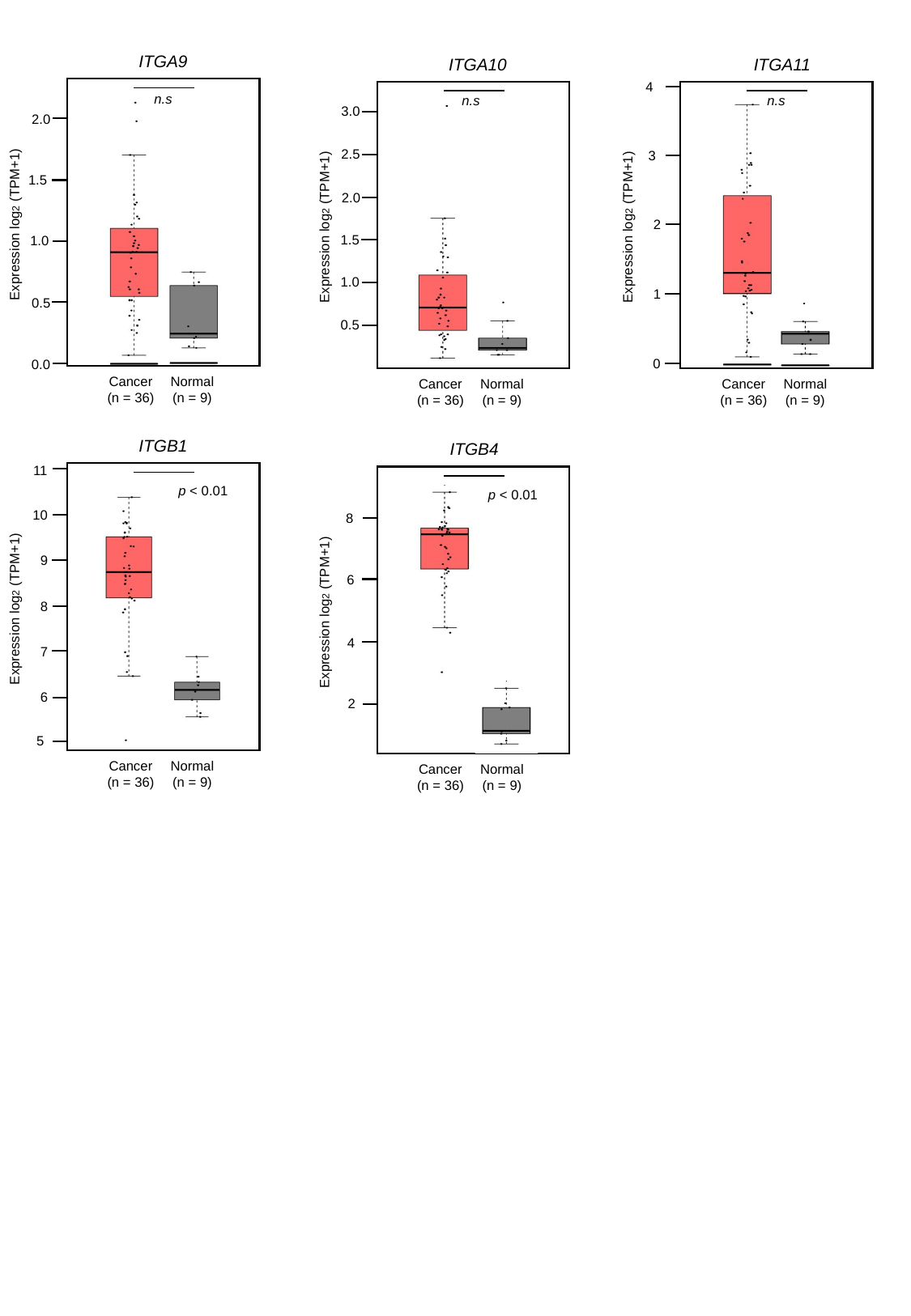

ITGA9
2.0
1.5
Expression log2 (TPM+1)
1.0
0.5
0.0
Cancer
(n = 36)
Normal
(n = 9)
n.s
ITGA10
3.0
2.5
2.0
Expression log2 (TPM+1)
1.0
0.5
Cancer
(n = 36)
Normal
(n = 9)
n.s
1.5
ITGA11
4
3
2
Expression log2 (TPM+1)
1
0
Cancer
(n = 36)
Normal
(n = 9)
n.s
ITGB1
11
p < 0.01
10
9
8
Expression log2 (TPM+1)
7
6
5
Cancer
(n = 36)
Normal
(n = 9)
ITGB4
p < 0.01
8
6
Expression log2 (TPM+1)
4
2
Cancer
(n = 36)
Normal
(n = 9)
