# Supplementary figures and images for: Molecular Pathogenesis and Regulation of the miR-29-3p-Family: Involvement of ITGA6 and ITGB1 in Intra-Hepatic Cholangiocarcinoma
Source: Cancers (Basel). 2021 Jun 4;13(11):2804. doi: 10.3390/cancers13112804 (PMC8200054; doi:10.3390/cancers13112804)

## Slide 1
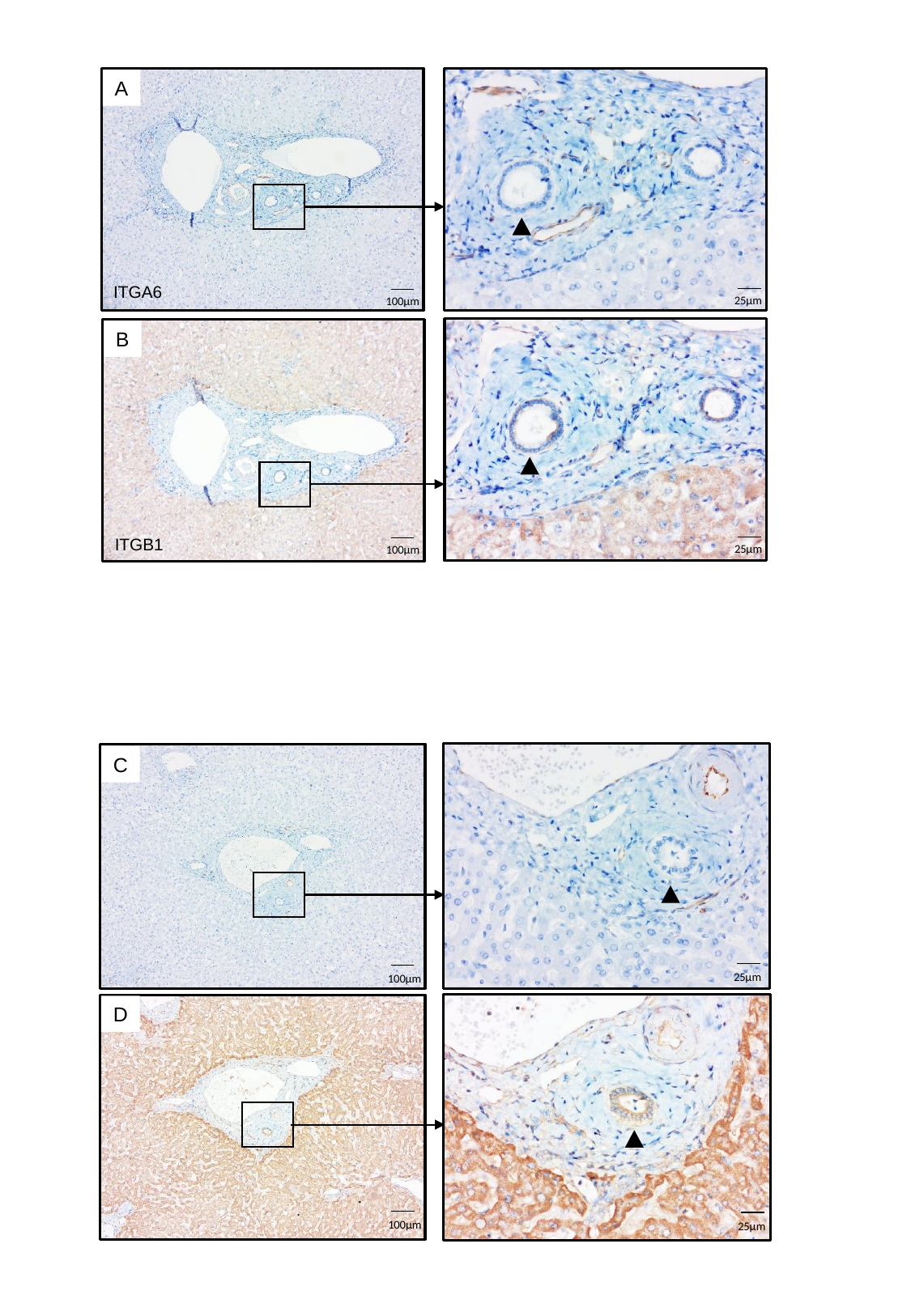

A
ITGA6
25µm
100µm
B
ITGB1
25µm
100µm
C
25µm
100µm
ITGB1
D
100µm
25µm

Supplement: Supplementary file 1 [file cancers-13-02804-s001.zip › supplementary files/Figure S1.pptx]

## Slide 1
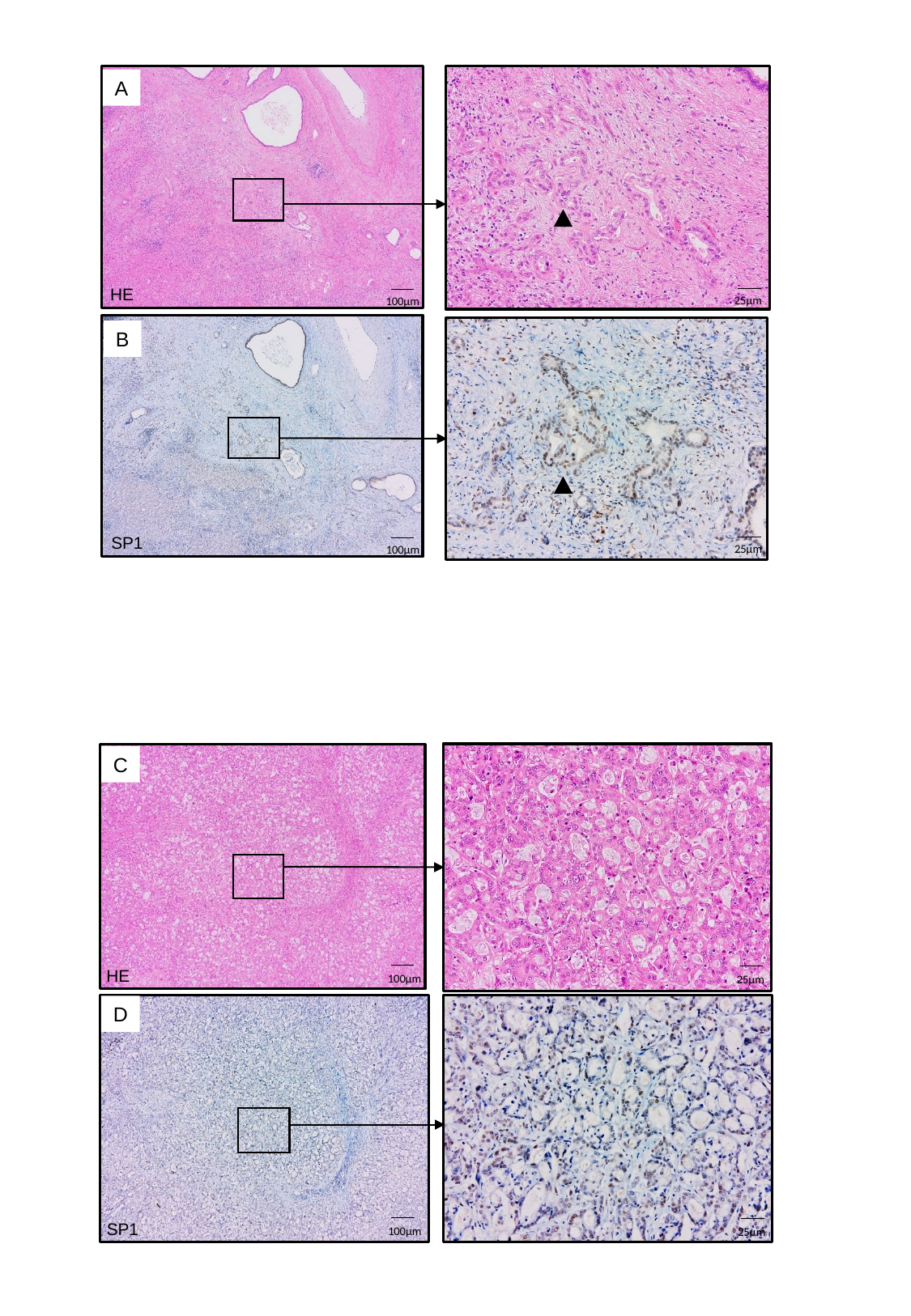

A
HE
25µm
100µm
B
SP1
25µm
100µm
C
HE
100µm
25µm
D
SP1
100µm
25µm

Supplement: Supplementary file 1 [file cancers-13-02804-s001.zip › supplementary files/Figure S7.pptx]
